# Period-Tuned a-C/a-C:H Multilayer DLC Coating for Tribocorrosion Protection of HSLA-100 Steel

**DOI:** 10.3390/nano15221704

**Published:** 2025-11-11

**Authors:** Tong Jin, Ji-An Feng, Yan Huang, Zhenghua Wu, Xinyi Guo, Kailin Zhu, Wei Dai, Yansheng Yin, Hao Wu

**Affiliations:** 1 School of Electromechanical Engineering, Guangdong University of Technology, Guangzhou 510006, China; 2112301036@mail2.gdut.edu.cn; 2 Guangdong Key Laboratory of Materials and Equipment in Harsh Marine Environment, School of Ocean Engineering, Guangzhou Maritime University, Guangzhou 510725, China; jafeng039@hotmail.com (J.-A.F.); yanhuang1804@outlook.com (Y.H.); wzh13423945193@163.com (Z.W.); xinyiguo089@163.com (X.G.); 15119064421@163.com (K.Z.); ysyin@shmtu.edu.cn (Y.Y.)

**Keywords:** diamond-like carbon, friction, corrosion, modulation period, arc-ion plating

## Abstract

By alternately depositing hydrogen-free amorphous carbon (a-C) and hydrogenated amorphous carbon (a-C:H) nanolayers on HSLA-100 steel through arc-ion plating, multilayer diamond-like carbon (DLC) architectures were engineered, with the modulation period adjusted from 1 to 10 cycles. SEM and Raman spectroscopy served as the analytical tools for characterizing the microstructure. For assessing key functional behaviors, nanoindentation was used to test mechanical properties, dry-sliding tribometry and in-situ tribocorrosion tests targeted tribological and tribocorrosion performance, and polarization tests focused on corrosion resistance. Introducing C_2_H_2_ increased the sp^3^ fraction and hardness relative to pure a-C. The ten-period film (S5) yielded the highest H/E (0.0767) and H^3^/E^2^ (0.171), reflecting the best hardness–toughness synergy. All coatings lowered the dry friction coefficient to 0.08–0.10 and cut wear by more than 1 order of magnitude versus the substrate; the ten-period film (S5) showed the minimum dry wear rate (1.39 × 10^−7^ mm^3^·N^−1^·m^−1^) and tribocorrosion wear rate (4.53 × 10^−7^ mm^3^·N^−1^·m^−1^) in 3.5 wt% NaCl. The superior performance is due to interlayer interfaces that dissipate stresses, arrest crack propagation, and block electrolyte ingress through defects. These findings indicate that the rational stacking of a-C/a-C:H significantly improves the tribological and tribocorrosion resistance of HSLA-100, providing a reliable protective approach for components used in marine services.

## 1. Introduction

With the continuous growth in the global demand for energy and raw materials, the extraction of deep-sea oil and gas as well as the collection of offshore renewable energy have emerged as strategic priorities for safeguarding energy supplies and advancing sustainable development [1,2]. Station-keeping with high accuracy is essential for floating production, storage, and offloading (FPSO) units and semi-submersible floating wind platforms; their mooring systems—most notably high-strength chains and supporting hardware—must endure reciprocating sliding wear caused by waves, currents, and wind loads throughout their operational lifespan and meanwhile resist corrosion triggered by seawater and bio-fouling. In marine environments, wear and corrosion frequently co-occur and interact synergistically, giving rise to an irreversible tribocorrosion failure mode that severely shortens fatigue life and compromises the structural integrity of critical components [3,4,5,6,7,8,9]. High-strength low-alloy steel (HSLA-100), prized for its high specific strength and excellent weldability, is therefore the material of choice for deep-sea mooring chains and deck equipment. However, under tribocorrosive attack in 3.5 wt% NaCl seawater, the fatigue life of HSLA-100 has been reported to drop by more than 60%, with crack initiation occurring earlier and propagation rates accelerating markedly [10,11,12,13,14]. Therefore, developing functional coatings that combine high toughness, low defect density, and exceptional corrosion resistance is critical for enhancing the tribocorrosion resistance of HSLA-100 steel [7,9,15,16,17,18,19]. Diamond-like carbon (DLC) coatings serve as prevalent protective overlays thanks to their superior wear and corrosion resistance, as well as inherent self-lubricating properties [1,20,21,22,23]. DLC coatings are broadly categorized into hydrogen-free amorphous carbon (a-C) and hydrogenated amorphous carbon (a-C: H). The former attains a hardness of 18–25 GPa and superior load-bearing capacity owing to its high sp^3^ content, whereas the latter achieves markedly lower corrosion current densities through hydrogen passivation of defects, albeit at a modest hardness of 10–18 GPa. When hydrogen content is excessively high, hydrogen atoms extensively intercalate between carbon–carbon bonds to form C-H bonds. An overabundance of C-H bonds hinders carbon atoms from forming sp^3^ cross-linked structures, instead promoting the aggregation of sp^2^ bonds. This ultimately leads to a decrease in sp^3^ content and material softening—the more commonly observed “hydrogenation-induced hardness reduction” phenomenon. Conversely, incorporating only a small amount of hydrogen removes unstable sp^2^ structures, forcing the remaining carbon atoms to rearrange into more stable sp^3^ bonds (diamond-like structures). This increases sp^3^ content and enhances hardness.

The main difference between the two is the presence of hydrogen, which leads them to differ in hardness, stability and application scenarios. Guan et al. [24,25] highlighted the advantages of using DLC coatings for solid lubrication in wet conditions, as they were found to significantly reduce wear and friction coefficients. Wu et al. [8] demonstrated that lower residual stress prevented corrosion channel formation in DLC coatings, significantly improving their corrosion resistance. The high graphitic structure content also effectively resisted micro-cracking caused by shear stress. Zhang et al. [5] improved DLC coating tribocorrosion performance via carbon bond and microstructure adjustments. Zhang et al. [26] subjected a CoCrMo alloy surface to the application of both hydrogenated and hydrogen-free diamond-like carbon coatings. The study revealed that the friction properties of DLC coatings are predominantly influenced by factors such as the lubrication medium, the interacting material, and the resultant transfer film. Moreover, they discovered that the a-C:H coatings exhibited reduced sensitivity to aqueous environments when compared to the hydrogenated DLC coatings. Ye et al. [27] applied unbalanced magnetron sputtering to apply a-C:H films to 304 L stainless steel. The resulting coatings exhibited improved tribocorrosion resistance in seawater compared to the 304 L substrate. The volume loss of the a-C:H coating in the tribocorrosion experiments was mainly dominated by pure mechanical wear. Zhang et al. [6] and others comparatively investigated the frictional corrosion performance of thermally sprayed WC-based ceramics/cemented carbides with hydrogenated diamond-like coatings and hydrogen-free diamond-like coatings. WC-based ceramics demonstrated enhanced tribocorrosion resistance in 3.5 wt% NaCl solution regardless of hydrogen presence.

Hydrogenated DLC coatings demonstrate excellent tribocorrosion performance even under minimal loads, thanks to their exceptional hardness and corrosion resistance, but suffer at heavy loads due to high brittleness and residual stresses, which lead to catastrophic delamination and protective degradation of the coatings. While a-C:H coatings offer higher hardness and superior corrosion resistance compared to a-C coatings, their high hardness leads to significant brittleness—making them prone to cracking and spalling under impact or sudden loads—whereas a-C coatings exhibit better load-bearing capacity. Meanwhile, the architecture of a coating is crucial to its shielding performance. For example, DLC coatings fabricated via techniques such as HiPIMS, magnetron sputtering, and cathodic multi-arc evaporation often contain structural imperfections like voids and oversized particles. These defects reduce the coatings’ corrosion resistance and, in turn, diminish their protective effectiveness in harsh corrosive environments [28,29,30]. Overall, a-C:H coatings have advantages in terms of hardness and corrosion resistance, while a-C coatings perform better in load-bearing capacity. Optimizing coating architecture (i.e., reducing defects) represents a key direction for enhancing the performance of various coatings under complex working conditions.

Many literature sources have shown that alternating multilayer structures can significantly reduce residual stresses while reducing penetration-type defects to prevent more corrosive solutions from entering the substrate. Ma et al. [31] successfully fabricated CrN monolayers and CrN/Al nanolayer coatings on F690 steel substrates using reactive magnetron sputtering technology. The study revealed that this nanolayer structure exhibits a significant ‘pore-sealing effect’, which effectively prevents corrosive substances from reaching the substrate surface by penetrating the coating. Through the periodic modulation of bias voltage in a filtered cathodic vacuum arc (FCVA) apparatus, Wei et al. [32] fabricated multilayered films with varying sp^3^ concentrations and in doing so produced tetrahedral amorphous carbon (ta-C) thin coatings with adjustable sp^3^ content. Their findings indicate that this multilayer architecture significantly diminishes residual stress levels in ta-C coatings while effectively curbing the delamination tendency frequently observed in monolithic ta-C configurations. However, systematic investigations into alternating hydrogenated/non-hydrogenated DLC (a-C/a-C:H) gradient multilayers and their synergistic protection mechanisms remain scarce.

However, as shown in Table 1, there is a lack of studies on the friction and corrosion properties of combining hydrogenated and hydrogen-free DLC coatings into multilayer coatings, which differ in friction and corrosion properties due to environmental effects. We employed periodic tuning of a-C/a-C:H coatings on HSLA-100 steel via arc ion plating to simulate marine friction corrosion in a 3.5 wt% NaCl environment, with 10 cycles identified as optimal. Compared to prior studies, this coating exhibits relatively lower thickness, requiring minimal target material, while the arc ion plating process enables significantly faster deposition rates.

## 2. Materials and Methods

### 2.1. Coating Preparation

In this experiment, five sample categories (S1, S2, S3, S4, and S5) were deposited; among them, S3, S4, and S5 are multilayer coatings, which consist of a-C (S1)/a-C:H (S2) bonding with 1, 5, and 10 cycles respectively. To boost the adhesion of the DLC layer to the underlying substrate, a titanium transition layer was first added to the base material—while the Ti layer was kept at roughly 300 nm in thickness, the DLC coating was maintained at approximately 700 nm. Before deposition, the HSLA-100 substrate was meticulously prepared through cutting and polishing with 2000 mesh sandpaper, followed by ultrasonic cleaning in a metal washing solution and ethanol for 30 min each. With the substrate fully prepped, it was then placed into the deposition chamber. As our target materials, we utilized high-purity graphite (99.99%) and titanium (99.9%). Prior to kicking off the deposition process, the chamber was pumped down to create a vacuum environment with pressure below 5.0 × 10^−3^ Pa. To top things off, the substrate underwent a glow cleaning treatment for 30 min, which involved introducing 200 sccm of argon gas at a bias pressure of 1.5 Pa while applying a −900 V voltage. Then ion etching was performed by passing 150 sccm Ar gas at a bias pressure of −600 V at a After the ion etching was completed, the substrate was bombarded with 200 sccm of Ar gas using DC magnetron sputtering technology at a bias pressure of −600 V for 5 min to minimize the residual oxygen on the surface of the substrate, which was then followed by depositing the Ti transition layer for 25 min at a bias pressure of −100 V with a power of 2 kW. a-C and a-C:H coatings were prepared using arc ion plating technology. For the a-C coating, 90 sccm Ar gas was introduced under a −50 V bias for 22 min. For the a-C:H coating, 90 sccm Ar gas and 10 sccm C2H2 were introduced under a −50 V bias for 21 min. The working gas pressure was 0.2 Pa for both coatings, and the arc current was 100 A. The substrate temperature naturally increases from 80 °C under the influence of the arc ion plating process. A direct current arc current of 100 A is employed, with the voltage stabilizing at 21 V. The distance between the magnetron target and the substrate is 10 cm, while the distance between the arc target and the substrate is 20 cm. The coil current is 3A, and the voltage is 20 V.

**Figure 1:**
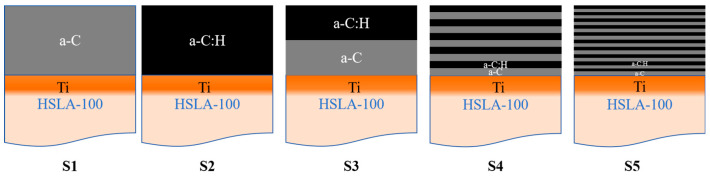
Coating structure schematic diagram.

By controlling whether acetylene is introduced to achieve the deposition of a-C and a-C:H multilayer coatings, we place the harder a-C:H on the surface layer to expect better wear resistance. Due to the switching of acetylene will have an effect on the rate of deposition, so each stage of the deposition time of the 10 L is added 5 s to ensure that the coating thickness remains consistent. thickness is consistent. The structure of the coating is shown in Figure 1 and Table 2 for the schematic diagram and deposition parameters, respectively.

### 2.2. Coating Characterization

We took a closer look at the diamond-like carbon coating’s cross-sectional morphology using scanning electron microscopy(Helios 5 CX, Thermo Fisher Scientific, Massachusetts, USA). To get the lowdown on how carbon atoms were bonding within the coating, we employed Raman spectroscopy (inVia Qontor, Renishaw plc, London, UK)with a laser wavelength fixed at 532 nm, covering the spectral range from 500 to 2000 cm^−1^. After importing the data, the user-defined settings in peak analysis were used to flatten the baseline between 500–1000 cm^−1^ and 1750–2000 cm^−1^. Subsequently, manual peak detection was performed to add the D peak and G peak at 1350 cm^−1^ and 1580 cm^−1^, respectively. Finally, Gaussian fitting was controlled to achieve convergence. For the nanoindentation tests (TTX-NHT2, Anton Paar, Graz, Austria), we used specialized equipment applying a 2 mN load, assuming a Poisson’s ratio of 0.25 for our analysis. The maximum indentation depth of the coating is approximately 55 nm. It does not exceed 10% of the coating thickness. At least three repetitions of the test were performed with 12 points selected for each sample. The residual stress in the coating is measured using a coating residual stress testing instrument (Supro Instruments FST-1000, Shenzhen, China). The curvature r of the silicon wafer is measured prior to coating deposition. Subsequently, the curvature ***k*** after coating deposition is measured. To ensure data accuracy, each sample group undergoes at least three tests, with the final value being the average. When ***r*** >> ***h***_S_ >> ***h_f_***, the Stoney formula can be applied:

(1) $$\sigma =\frac{E_{S}}{1-V_{S}}\times \frac{{h_{S}}^{2}}{{6h}_{f}}\times k$$

Here, ***E_S_*** and ***Vs***. denote the Young’s modulus and Poisson’s ratio of the substrate, respectively, while ***h_S_*** and ***h_f_*** represent the thicknesses of the substrate and coating. Measure the roughness of the sample and a 10 × 10 μm area at the center of each sample’s grinding mark using a white light interferometer.

### 2.3. Tribological Test

The friction properties of various samples were examined using a reciprocating tribometer (UMT Tribolab, Bruker, Massachusetts, USA) at room temperature. During the test, Φ6 mm silicon nitride balls were run with fixed specimens. The sliding test applied 2 N normal force with 3 mm stroke length, 5 Hz frequency, and 3600 s duration, with each measurement repeated at least three times. Once we wrapped up the testing phase, we utilized a white light interferometer to analyze the surface profile of the wear track, allowing us to pin down its cross-sectional area. From there, we crunched the numbers to determine the total wear volume by multiplying this area measurement by the complete length of the wear track.

### 2.4. Electrochemical Measurements

The samples were subjected to a series of tests to ascertain their kinetic potential polarization, a process conducted with the aid of an electrochemical workstation (PMC1000-AT, AMEETK, Pennsylvania, USA). The electrochemical setup employed a saturated calomel electrode as the reference, with a platinum wire serving as the counter electrode, while the test material itself functioned as the working electrode. This working electrode boasted a circular configuration with a diameter of 1.5 cm. Before running any electrochemical analyses, the specimen was immersed in synthetic seawater for a ten-minute period to reach a stable open-circuit potential. Polarization measurements were then carried out across a voltage range extending from −0.25 V to 0.5 V, employing a scan rate of 1 mV/s. Once these experiments wrapped up, both the corrosion potential (E_corr_) and corrosion current density (i_corr_) were determined from the resulting Tafel plots.

### 2.5. Tribocorrosion Test

The tribocorrosion properties of various samples were examined using a reciprocating tribometer (UMT Tribolab, Bruker, Massachusetts, USA). During the test, Φ5 mm silicon nitride balls were run with a fixed specimen immersed in 3.5% NaCl solution. For the electrochemical tests, the sample was employed to act as the working electrode, while two other distinct electrodes—an Ag/AgCl one for reference and a platinum wire mesh for counter purposes—were used in matching roles. The sliding test applied 2 N normal force with 3 mm stroke length, 5 Hz frequency, and 3600 s duration, and at least three repetitions were performed for each measurement. The test was divided into three parts: After stabilizing the open-circuit potential for 10 min, sliding friction was initiated for 3600 s. The immersion continued for a further 10 min after the completion of the friction test to stabilize the open-circuit potential. With testing wrapped up, we employed a white light interferometer to analyze the surface profile and determine the cross-sectional area of the wear track. Next, we multiplied this cross-sectional area by the length of the wear track to calculate the overall wear volume. The wear of silicon nitride balls is negligible within the measurement resolution. To top it all off, scanning electron microscopy (Helios 5 CX, Thermo Fisher Scientific, Massachusetts, USA) was brought into the mix to take a closer look at the surface morphology of the wear marks.

## 3. Results and Discussion

### 3.1. Characterization of DLC Coatings

**Figure 2:**
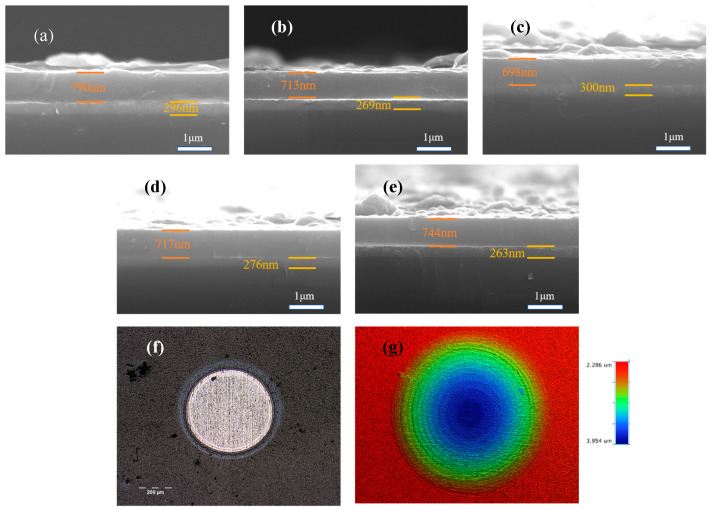
The SEM cross-sections (**a**–**e**) represent the microscopic morphology of S1 to S5. (**f**) Optical morphology of S4 sample ball pits and (**g**) 3D profile.

Figure 2 lays bare the cross-sectional architecture of the deposited DLC coatings. Every sample reveals a compact, consistent internal structure devoid of microscopic imperfections or fractures, with each forming a robust interface with the underlying substrate. The Ti transition layers for specimens S1 through S5 measure 296, 269, 300, 276, and 263 nanometers, while their corresponding DLC coatings come in at 790, 713, 698, 717, and 744 nanometers thick, respectively. The coating thickness varies due to the fact that during the period of energizing and stopping of the energizing acetylene will have an effect on the deposition rate, the coating thicknesses varied. Nevertheless, the negligible thickness variations between the samples negate the impact of thickness on friction and corrosion characteristics. Figure 2f,g show the optical morphology of the ball pits and the 3D contours of the ball pits taken by the white light interferometer for the S4 sample, respectively, which shows that the ball pits have a clear multilayer delamination phenomenon, which can indicate that multilayer coatings have been successfully deposited.

**Figure 3:**
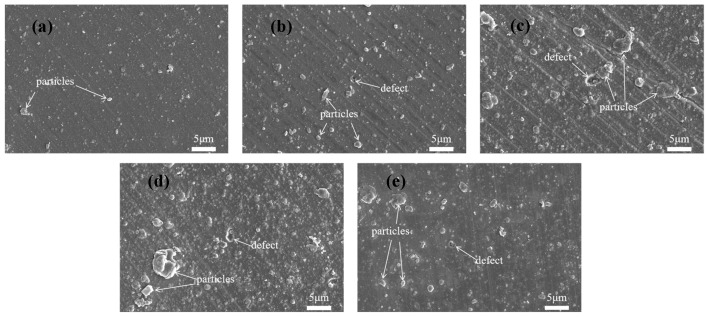
The morphological appearances of the surfaces of (**a**) S1, (**b**) S2, (**c**) S3, (**d**) S4, and (**e**) S5.

As shown in Figure 3, the surface of S1 is smooth and dense, but there are some large particles of carbon; the particles on the surface of S2 are larger and more numerous compared to the surface of S1, which is an effect of the passage of acetylene; the particles on the surfaces of S3 and S4 become larger and there are more surface defects, and the thickness of the coatings and the deposition process of each layer affects the generation of large particles when a-C and a-C:H coatings are combined to form a multilayer structure. In multilayer structures, the deposition process of each layer of material may be affected by the previous layer of coating, resulting in the gradual accumulation of particles during deposition and the formation of larger particles. As shown in Table 3, the surface roughness of the samples is consistent with that observed by SEM.

**Figure 4:**
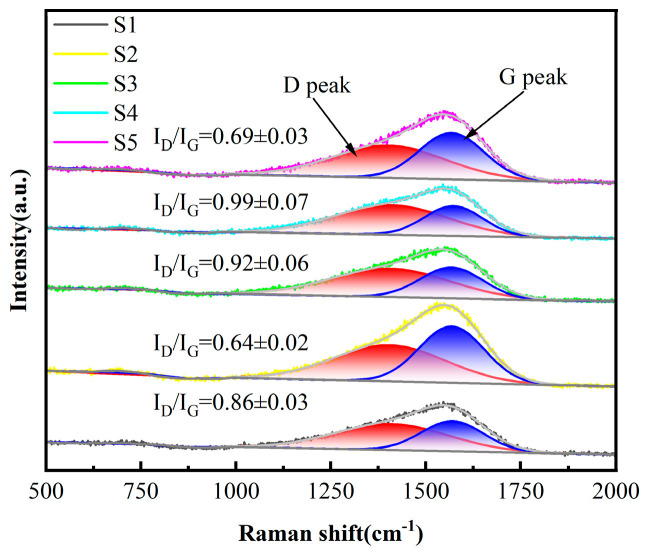
The Raman spectra of the five samples. (*n* = 3).

As presented in Figure 4, the Raman spectra of the deposited DLC coatings have similar traits, as displayed in Figure 4. A broad, asymmetric peak is observed in the 1000–1800 cm^−1^ range, which points to an asymmetric carbon structure; this spectrum comprises two Gaussian peaks: the D peak at around 1350 cm^−1^ (primarily due to defects in the C atom lattice) and the G peak at around 1580 cm^−1^ (a telescopic vibration within the sp^2^ hybridization surface of carbon atoms) [33,34]. The D and G peak parameters were extracted utilizing Origin 2025 software. Further examination of the Raman fitting outcomes indicates that the D-peak to G-peak intensity ratio (I_D_/I_G_) is capable of offering significant understanding of the DLC coatings’ structural characteristics. The relative amount of sp^2^ and sp^3^ bonding in the carbon film can be indicated by I_D_/I_G_ [32], and a larger I_D_/I_G_ indicates the existence of a greater quantity of defects or disordered structures within the carbon material. With the passage of C_2_H_2_, the value of I_D_/I_G_ decreases gradually (0.86~0.64), indicating that the doping of C_2_H_2_ favors the formation of sp^3^ bonding. The higher I_D_/I_G_ of S3 and S4 relative to that of S1 and S2 suggests that there are more defects or disordered structures in the coatings, which aligns with the findings of SEM. This is due to the accumulation of defects during the deposition process. Each layer introduced during deposition introduces intrinsic defects (such as vacancies and impurities). A multilayer structure corresponds to multiple deposition cycles plus multiple interface formations, where defects accumulate at both the layers and interfaces. In contrast, a monolayer film requires only a single deposition cycle, resulting in fewer defect sources. The reduction in I_D_/I_G_ for S5 samples with more layers is attributed to the extremely short deposition time per layer, which prevents defects from forming and accumulating sufficiently.

**Figure 5:**
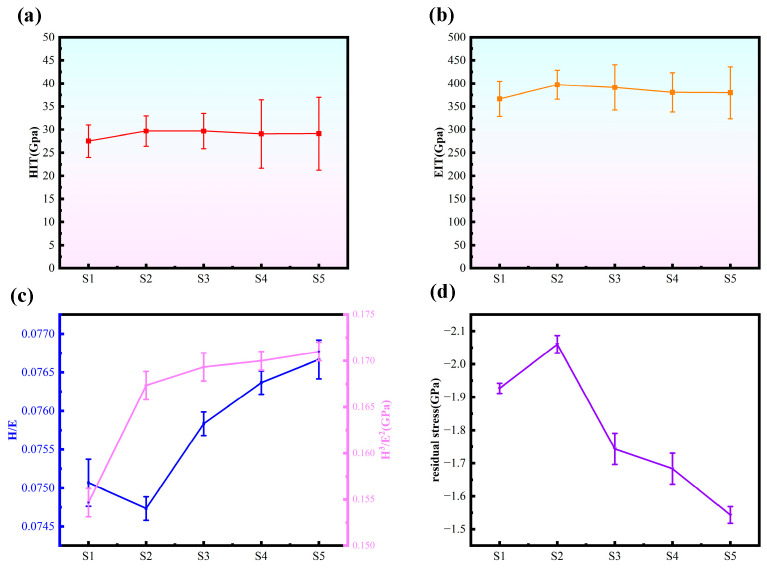
(**a**) The hardness of the five samples, (**b**) the elastic modulus of the five samples, (**c**) the H/E ratio of the five samples, and the H^3^/E^2^ ratio of the five samples, (**d**) the residual stress of the five samples (*n* = 3).

As depicted in Figure 5, the hardness and elastic modulus of each sample were evaluated. When compared to S1 (27.5 GPa, 366.6 GPa), S2 exhibited notable improvements, boasting a hardness of 29.7 GPa and an elastic modulus of 397.1 GPa. The multilayer coatings—S3, S4, and S5—which combined a-C and a-C:H layers, demonstrated slightly reduced values compared to S2; however, they still surpassed the performance of S1 samples. This trend aligns neatly with straightforward mixing principles. H/E and H^3^/E^2^ reflect the material’s resistance to wear and deformation, where higher values correspond to a higher resistance of the material to wear and deformation [21,35,36,37,38]. As can be seen in the figure, after the passage of acetylene, S2 than S1 samples H/E and H^3^/E^2^ are improved; the passage of acetylene can improve the wear resistance of the a-C layer, the doping of C_2_H_2_ is conducive to the development of sp^3^ bonding, and the increase in the content of sp^3^ makes the samples have better wear resistance. The multilayer structure improves H/E and H^3^/E^2^ compared with single layer, which implies that the multilayer structure contributes to enhancing the wear resistance characteristics of the coatings, as it lowers the internal stresses of the coatings. Meanwhile, for the multilayer coated samples, as the number of cycles increases, H/E and H^3^/E^2^ first rise and then fall. 10 cycles of S5 samples with the largest H/E (0.0767) and H^3^/E^2^ (0.171), which have the best mechanical properties. which has the best mechanical properties. Ma et al. [31] deposited CrN/AlN nanolayer coatings with H/E and H^3^/E^2^ values of 0.07 and 0.10, respectively; Li et al. [39] deposited TiCx/DLC coatings with an H^3^/E^2^ value of 0.157; Beliardouh et al. [40] deposited Ta/ZrN multilayer coatings exhibited a maximum H^3^/E^2^ value of 0.151. Compared to these multi-layer coatings, this study still exhibits a higher H^3^/E^2^ ratio, superior resistance to plastic deformation, and enhanced wear resistance.

As shown in Figure 5d, the residual stress in S2 increased compared to S1. This is attributed to the incorporation of C_2_H_2_, which elevated the sp^3^ bond content in the coating—a finding corroborated by Raman analysis. The increased sp^3^ content enhanced the coating’s hardness while simultaneously raising the internal stress in S2. The internal stress in multilayer coatings is consistently lower than that in monolayer coatings. The interface between hard and soft layers forms a flexible transition zone that both prevents the propagation of microdefects (such as cracks and atomic dislocations) within the hard layer and provides a stress release pathway through slight deformation of the soft layer, thereby reducing stress accumulation within the coating. As the number of cycles increases, the quantity of interfaces grows, leading to a corresponding decrease in internal stress within the multilayer coating.

### 3.2. Dry Friction Behavior

**Figure 6:**
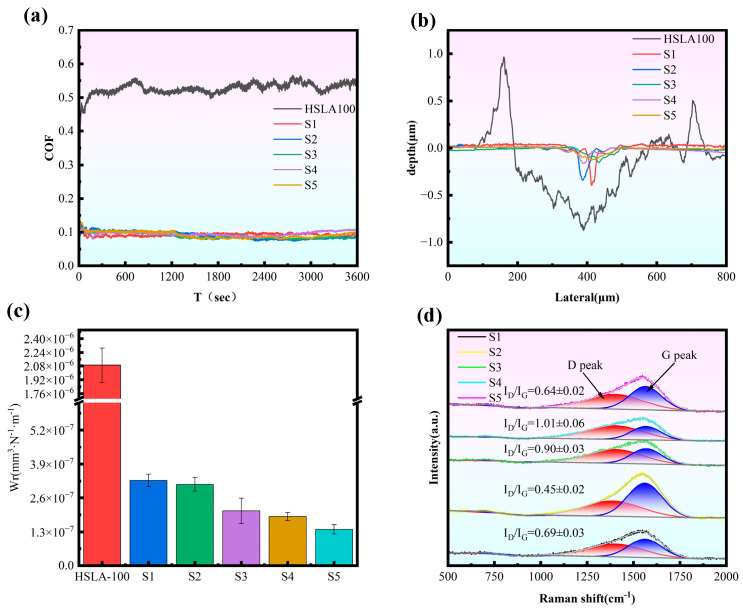
(**a**) The friction coefficient curve observed throughout the dry friction testing process, (**b**) the wear track depth recorded following completion of the dry friction test, (**c**) the wear rate determined after the dry friction experiment concluded, and (**d**) the Raman spectra analysis of wear scars for all five samples tested under dry friction conditions. (*n* = 3).

Figure 6a presents the friction coefficient profiles for each specimen plotted against sliding time. Notably, the HSLA100 substrate demonstrates a friction coefficient exceeding 0.4, accompanied by significant oscillations throughout the testing period. On the other hand, the coated sample exhibits a remarkably stable curve, with friction coefficients maintaining a tight band between 0.08 and 0.1. This substantial reduction compared to the bare substrate points to superior lubricating properties. Figure 6b shows the cross-section of the wear trajectory. The HSLA100 substrate has the widest wear trajectory, producing a high volume of debris and exhibiting poor wear resistance. On the contrary, the wear trajectories of the coated samples become narrower, in which the S1 and S2 samples have narrow and deep wear traces, while the multilayer coated samples (S3, S4, S5) have wide and shallow wear traces, and the friction forms of the single layer and multilayer are different.

As shown in Figure 6c and Table 4, the wear rate of every individual sample was compared. The coated samples’ wear rates were all reduced by at least one order of magnitude compared to the HSLA100 substrate, indicating that the coatings provide excellent protection. The multilayer coatings had a lower wear rate than the single-layer coatings, and this decreased with an increase in cycles. The S5 samples had the lowest wear rate, which was attributed to the release of internal stresses by the softer layers and multilayer interfaces, which hindered the emergence and expansion of microcracks. This reduces the generation and attachment of brittle debris during sliding, as well as the groove effect. Therefore, the wear resistance of DLC films is not directly proportional to hardness. It is important to strike a balance between hardness, resistance to plastic deformation, and residual stress [41]. For the purpose of understanding the friction mechanism, Raman measurements were carried out on the wear regions. Figure 6d displays the Raman spectral analysis of the worn coating surfaces. After applying Gaussian fitting, the ID/IG ratio—calculated as the area proportion between the D and G peaks—was determined. Shifts in this ID/IG ratio can shed light on the underlying friction processes. The ID/IG values decreased from 0.86 to 0.64 for sample S1 and from 0.69 to 0.45 for sample S2 within the wear tracks. During the wear process, the coating surface undergoes abrasion or compaction, which leads to the reduction of non-ordered carbon atoms or defects and the structure becomes denser and more ordered, resulting in a decrease in the I_D_/I_G_ ratio. The I_D_/I_G_ ratio of the multilayer coating remains essentially unchanged. Meanwhile, as shown by the friction center roughness in Table 3, the multilayer coating exhibits a smaller increase in scratch roughness compared to the monolayer coating, indicating superior deformation resistance. The multilayer structure design facilitates stress release during friction, thereby enhancing wear resistance.

**Figure 7:**
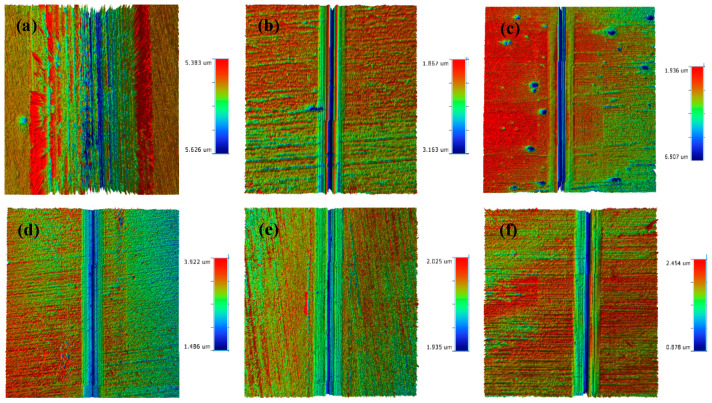
The dry friction 3D profiles of HSLA100 substrate, S1 to S5 are (**a**–**f**).

The morphology of the abrasion marks on the HSLA100 substrate is shown in Figure 7a, which shows that there are many abrasive grooves as well as adhesive bonds in the abrasion mark area, and thus the wear mechanism of the HSLA100 substrate is mainly abrasive and adhesive wear. Only abrasive grooves were observed in the coated samples and no obvious adhesive bonds were observed. The coated samples mainly exhibited abrasive wear, which was attributed to the lubricity of the DLC coatings themselves. This resulted in a significant reduction in adhesive wear.

### 3.3. Tribocorrosion Behavior

**Figure 8:**
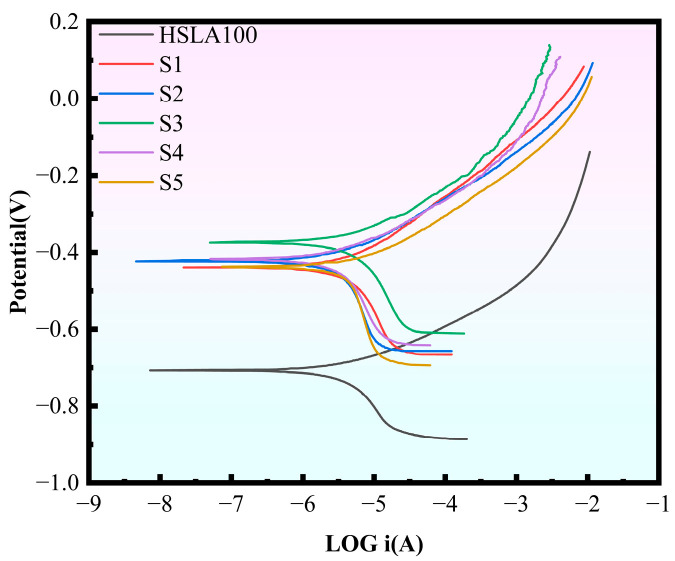
Polarization curves for the six samples.

To evaluate how well the HSLA100 substrate and multilayer DLC coatings hold up against corrosion in a 3.5 wt.% NaCl solution, we ran polarization curve tests on these specimens. Figure 8 displays the kinetic potential polarization curves for these samples when immersed in the 3.5 wt% NaCl solution Table 5 displays the corrosion potential (E_corr_) as well as the corrosion current density (i_corr_). All the samples showed active dissolution behavior in the anodic polarization region, which indicates that no passivation film was generated for all the samples. The samples’ overall corrosion resistance is typically contingent on the magnitude of the corrosion current density, in general, a lower corrosion current density corresponds to enhanced corrosion resistance. The samples coated in the experiment were all less corrosive than the substrate, and the corrosion potentials were all shifted in the positive direction. This indicates that the coating weakens the corrosion tendency relative to the substrate, improves the corrosion resistance, and provides a good protective effect on the substrate. The S2 sample exhibits a diminished corrosion current density in comparison to the S1 sample, a phenomenon attributable to the elevated sp^3^ content of the S2 sample. The multilayer coating exhibits superior corrosion resistance compared to the single-layer coating, as it is possible to reduce penetration-type defects. Furthermore, the corrosion current density decreases with an increase in cycles.

**Figure 9:**
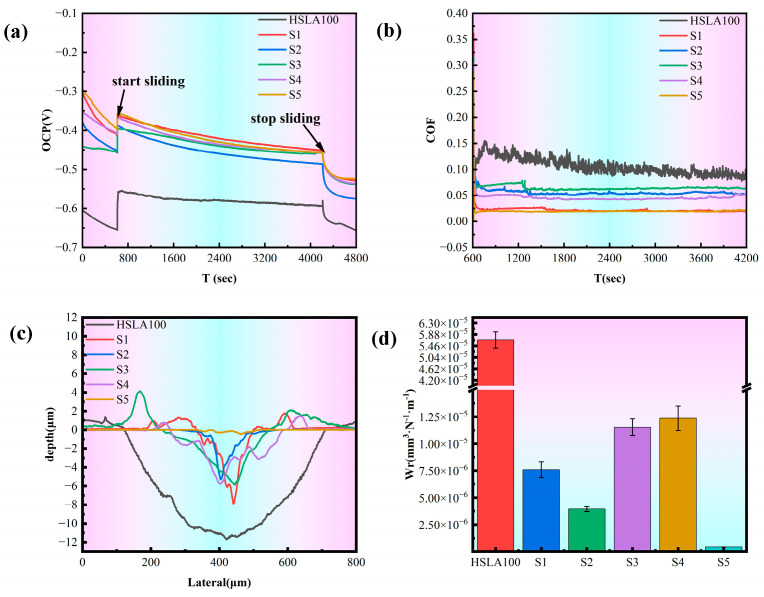
(**a**) The open circuit potential readings obtained during the tribocorrosion evaluation; (**b**) the friction coefficient profile observed throughout the tribocorrosion assessment; (**c**) the wear track depth measurements subsequent to the tribocorrosion procedure; (**d**) the average wear rate calculated following completion of the tribocorrosion test. (*n* = 3).

Figure 9a illustrates the open circuit potential (OCP) trajectory for both the HSLA100 substrate and DLC coating throughout the tribocorrosion process, offering a window into surface degradation characteristics. Upon initial loading with 2 N of static force, both specimens exhibited a positive shift in OCP readings. During testing, the potential exhibited a trend of sharply rising followed by a gradual decline. The steep increase in open-circuit potential (OCP) resulted from the stripping of the wear track surface, exposing a less active, pristine surface to the corrosive liquid. This potential difference created a corrosion microcell between the unworn and worn areas, accelerating corrosion in the worn region while slowing it in the unworn region. Simultaneously, the original potential of the unworn area continuously decreased during friction due to ongoing corrosion. The measured original potential represented the combined potential of the exposed areas. Since the corrosion microcell slowed corrosion in the unworn area, the overall original potential decreased slowly. Conversely, the open-circuit potential dropped sharply again after wear ceased. This trend resulted from the sustained rapid corrosion across the entire surface [42]. For coated samples, the OCP trend during the friction process was identical to that of the HSLA100 substrate, indicating that corrosive media still penetrated the coating to reach the substrate. However, compared to the HSLA100 substrate, the trend exhibited a positive shift, demonstrating the coating’s effective protective performance. Subsequent SEM results revealed exposed substrate in all coatings, explaining why the open-circuit potential values at the final stage differed only slightly (<0.03 V), it has stabilized at around −0.45 V.

Figure 9b illustrates the COF variations for HSLA100 substrate and DLC coating throughout tribocorrosion, with the HSLA100 COF declining from 0.15 initially to approximately 0.1, which has a certain lubricating effect of corrosion products and debris produced during the tribocorrosion process. And the COF of the coated samples are all stabilized between 0.01 and 0.06. The lowest friction coefficients of S1 and S5 were around 0.01, and the friction coefficients of S2 and S3 were around 0.06. This is due to the different friction characteristics of the a-C:H layer and the a-C layer itself in the solution, and the a-C layer has a better wettability in the solution, which makes the coating itself more lubricated in corrosive solutions [26]. Furthermore, it is noteworthy that passivating the dangling bonds of the a-C film is achievable. The solution environment contains a high concentration of chemically active molecules (e.g., water and oxygen), which passivate the dangling bonds on the contact surface. The friction coefficient is reduced by the weakening of the adhesion of the friction substitute due to the deactivation of the dangling bonds, which also results in good wear resistance [43,44]. S1 exhibits low surface energy, resulting in strong hydrophobicity. In solution, it readily forms a stable “liquid film lubrication layer,” reducing direct solid-solid contact and further lowering the coefficient of friction. S2/S3 possess higher surface energy due to C-H bonds, enhancing hydrophilicity. This causes the liquid film to rupture easily, increasing the probability of solid-solid contact. Even with higher hardness, it struggles to counteract the effects of adhesive friction, leading to a relatively high coefficient of friction.

**Figure 10:**
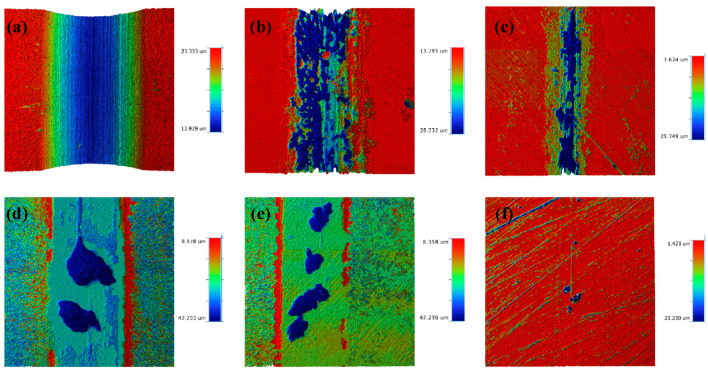
The tribocorrosion 3D profiles of HSLA100 substrate, S1, S2, S3, S4, and S5 are (**a**–**f**).

Figure 9c shows the tribocorrosion cross-section profile of each sample, in which the HSLA100 and single-layer coated S1 and S2 samples have different cross-section profiles at different locations due to the large peeling of the coating, and the whole cross-section is averaged by Vision software, and the figure shows the average cross-section of the whole abrasion marks. The coated samples have significantly less depth and width of abrasion marks than the HSLA100 substrate, and the coatings effectively protect the substrate. Figure 9d and Table 6 show the frictional corrosion wear rates of the samples. The findings indicate that a-C:H exhibits better frictional corrosion resistance than the a-C layer. However, the S3 and S4 coatings still wear out more quickly than the single-layer coating. S5 has the lowest wear rate of all the samples (4.53 × 10^−7^ mm^3^·N^−1^·m^−1^). This is way lower than the single-layer coating and way lower than the HSLA100 substrate. As illustrated in Figure 10, the wear rates of S3 and S4 exceed those of the single-layer coatings, primarily because the depth of pitting pits on S3 and S4 is far greater than that on the single-layer coatings, and the two also differ in their frictional corrosion mechanisms.

**Figure 11:**
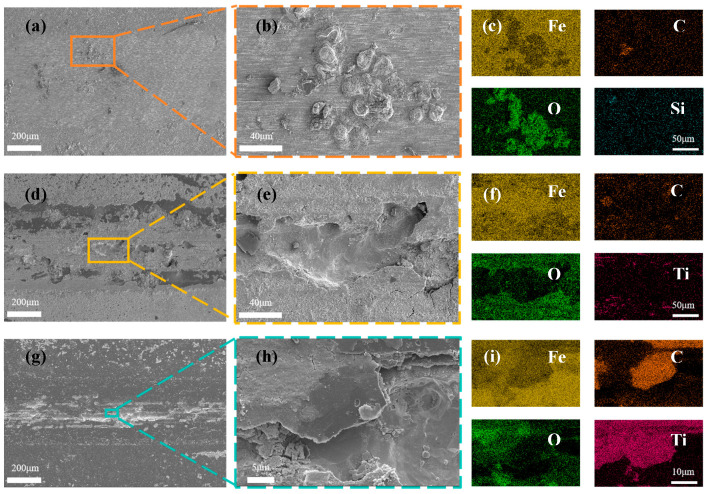
SEM images showing the wear surfaces of (**a**,**b**) HSLA100, (**d**,**e**) S1, and (**g**,**h**) S2 coatings post-sliding in 3.5% NaCl solution; and the corresponding EDS elemental maps for (**c**) HSLA100, (**f**) S1, and (**i**) S2 coatings.

For the purpose of exploring the frictional corrosion mechanisms of the respective samples, the abraded surfaces of the samples were characterized using an electron scanning microscope and EDS. The surface morphology of HSLA100 abrasion marks is shown in Figure 11a, and it can be seen from this that there are a lot of small grooves in the abrasion mark area, which is typical of abrasive wear. At the same time, some pitting areas are found in the abrasion scar area, as shown in Figure 11b, and some Fe oxide residues can be seen in the pitting area through EDS spectroscopy (Figure 11c), which indicates that the substrate itself is affected by abrasive wear in the frictional corrosion process of HSLA100, and at the same time, it is corroded in corrosive solution, and both of them work together and lead to an increase in the loss of the material. Figure 11d shows the surface morphology of the abrasion marks on the S1 sample. There are many continuous deep pits in the center of the abrasion marks and corrosion products are distributed on both sides. These corrosion products indicate that the substrate is corroded by the penetration-type defects of the coating in the frictional corrosion process, with the corrosion products being pushed to the outer side by the friction vice. Figure 11f shows the energy-dispersive X-ray spectroscopy (EDS) spectrum of Figure 11e. This spectrum indicates that there is only a trace amount of carbon residue on the surface exposed to the substrate. This suggests that the S1 sample experienced extensive flaking during frictional corrosion, thus losing its protective ability. Figure 11g shows the surface morphology of the abrasion marks on the S2 sample. It can be seen that, compared to the S1 sample, the flaking area in the center of the abrasion marks on the S2 sample is relatively small and is basically distributed along the middle line. The coating is basically intact on both sides of the abrasion marks, although there is still some C residue in the center. Figure 11i shows the energy spectrum of the EDS analysis of Figure 11h. It can be seen that the deep pits expose the substrate. However, there is still a large area of carbon residue next to the deep pits and the Ti transition layer remains intact. The S2 sample has a better protective effect than the S1 sample and doping with C_2_H_2_ improves the tribocorrosion resistance of the coating.

**Figure 12:**
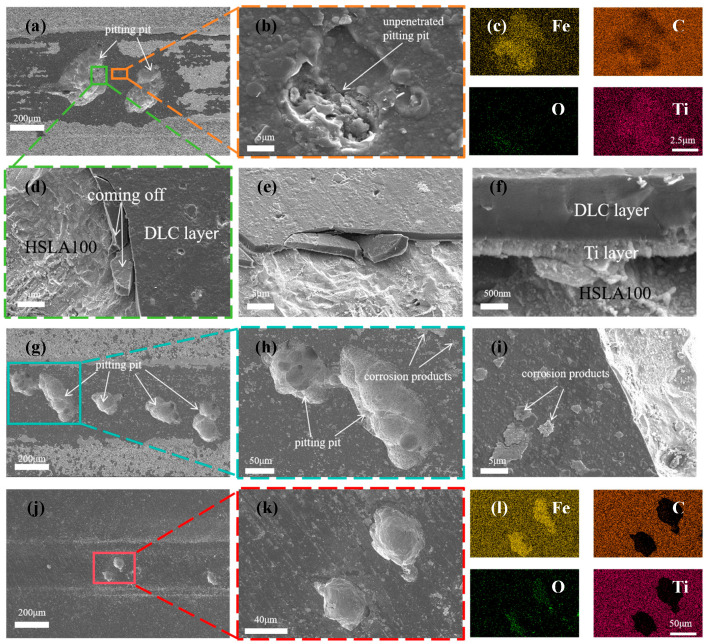
SEM images showing the wear surfaces of (**a**,**b**,**d**–**f**) S3, (**g**–**i**) S4, and (**j**,**k**) S5 coatings post-sliding in 3.5 wt% NaCl solution; along with the corresponding EDS elemental maps for S3 (**c**) and S5 (**l**) coatings.

**Figure 13:**
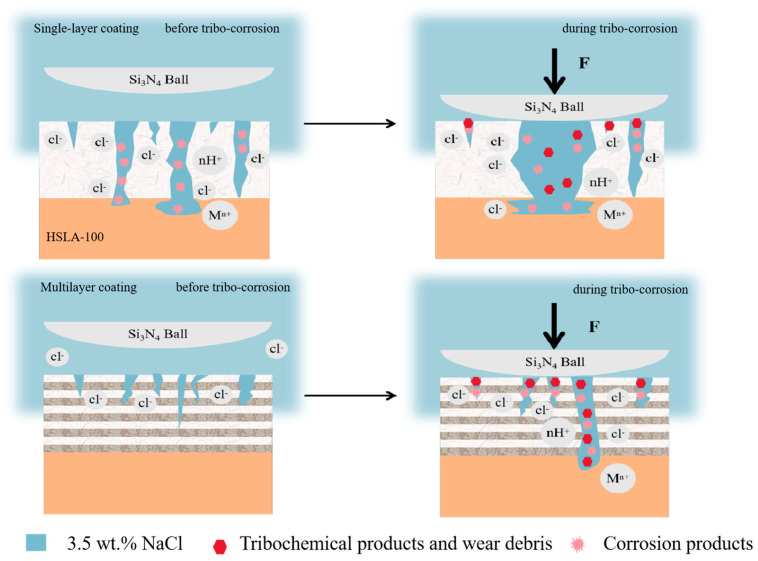
Schematic diagrams of the tribocorrosion mechanism of single-layer and multi-layer DLC coatings.

Figure 12a is a visual representation of the surface morphology of the abrasion marks of the S3 sample. S3 coating was not found to be continuous and flaked, unlike the single-layer coatings of S1 and S2. Only two pitting pits appeared, but these were very large (more than 200 µm in diameter). Some small pores were observed in the area of intact coatings (Figure 12b), which were not exposed to the substrate or the transition layer (Figure 12c). This demonstrates that the multilayer coating can effectively reduce the number of penetration-type defects. As demonstrated in Figure 12d, coating spalling is evident at the periphery of the pitting pit. By examining the cross-section (Figure 12e,f), it is evident that the coating spalling is initiated by the corrosion of the substrate. This results in the coating spalling due to inadequate bonding with the substrate. It can be shown that the mechanism of coating spalling is due to the corrosion solution through the coating penetration defects into the substrate, the substrate is corroded, the corrosion products generated through the defect′s outward penetration, and then pushed to the sides by the friction vice, the substrate is corroded, the coating in the friction because of insufficient bonding with the substrate to spalling. Figure 12g shows the surface morphology of the abrasion marks of the S4 sample, and there are several pitting pits in the abrasion mark area, but the diameter of the pitting pits is smaller than that of the S3 sample. Figure 12j shows the surface morphology of the S5 sample, in which there are fewer pits and the diameter of the pits is greatly reduced. Through the SEM results can be learned that the single-layer coating appeared large area spalling, while S3 and S4 only appeared large pitting pits, this is due to the excellent abrasion resistance of multilayer coatings lead to the reduction of microcracks while the multilayer structure to reduce the impact of penetration defects, the single-layer coatings in the friction process of the microcracks and penetration defects are more than the multilayer coatings in the tribocorrosion of early penetration defects at the beginning of the erosion of the corrosion of pitting pits, as time In the early stage of tribocorrosion, the penetration defects are corroded pitting, and with the growth of time, the pitting pits are getting bigger and bigger, and finally the neighboring pitting pits are connected together, and the phenomenon of single-layer coatings peeling off. The multilayer coating has fewer microcracks and penetration defects, so only a few large pitting pits appeared. The larger wear rate of S3 and S4 was due to the potential difference between the S3 and S4 pitting areas and the coating areas, which accelerated the corrosion in the pitting pits by forming microcells, resulting in a larger volume of loss in the pitting areas. With more cycles, the diameter of the pitting pits appearing in the multilayer coating is smaller. Coating tribocorrosion mechanism Figure 13: corrosive solution through the coating penetration defects and friction generated by the micro-cracks into the substrate, the substrate is corroded, the corrosion products generated through the defects outward infiltration, and then pushed to the sides by the friction side, at the same time, due to the substrate connected to the coating is corroded, the coating and the substrate is not enough to combine and spall off the formation of pitting pits, with the pitting pits of the growth of the growing and finally adjacent pitting pits are linked together As the pits grow larger and larger, and finally adjacent pits are connected together, the phenomenon of single-layer coating spalling occurs. In contrast, multilayer coatings exhibit fewer microcracks and permeation defects, resulting in only a small number of pitting cavities. This mechanism has been corroborated by relevant studies: Wan et al. [45] reported that a dense Al_2_O_3_ interlayer acts as an effective sealing layer, inhibiting charge transfer, diffusion of corrosive substances, and dislocation movement. Wei et al. [19] investigated the friction corrosion mechanism of Si/N-DLC, asserting that its superior corrosion resistance relates to the diffusion behavior of corrosion products and the formation of friction chemical products and wear debris, which block corrosion pathways. Ma et al. [31] demonstrated that nanostructured multilayer coatings exhibit a pronounced “pore-sealing effect,” making it difficult for corrosive solutions to penetrate the coating and corrode the substrate. Furthermore, due to galvanic coupling effects, the pitting depth at the substrate exposure points of multilayer coatings is greater than that of monolayer coatings.

## 4. Conclusions

The arc ion plating technique was used to successfully prepare single-layer a-C, a-C:H coatings and a-C/a-C:H multilayer coatings with different cycles on HSLA-100 steel substrates. This was achieved by controlling the through-flow of C_2_H_2_. Their friction and tribocorrosion mechanisms were investigated. The conclusions can be summarized as follows:

(1) Introducing C_2_H_2_ during arc-ion deposition increased the sp^3^ fraction, yielding harder and more corrosion-resistant DLC layers. The ten-period multilayer (S5) achieved the highest H/E (0.0767) and H^3^/E^2^ (0.171), evidencing the optimal hardness–toughness (elastic recovery–fracture resistance) balance.
(2) Under dry sliding, all coatings reduced the friction coefficient to ~0.08–0.10 and markedly lowered wear relative to HSLA-100; the ten-period film (S5) showed the minimum dry wear rate of 1.39 × 10^−7^ mm^3^·N^−1^·m^−1^.
(3) In 3.5 wt% NaCl tribocorrosion, multilayer architectures mitigated synergistic mechanical–electrochemical attack. S5 exhibited a wear rate of 4.53 × 10^−7^ mm^3^·N^−1^·m^−1^, which is approximately 16 times lower in comparison to S1 and two orders lower than the substrate, with corrosion pit size decreasing as the period number increased.
(4) These gains arise from interlayer interfaces that deflect and arrest crack propagation, dissipate stresses, and interrupt percolation pathways for electrolytes, thereby restricting corrosive species transport to the substrate.

This research features rapid deposition rates, significantly shortening the surface treatment cycle for large steel structures such as offshore platform pile legs, steel components of cross-sea bridges, and offshore pipelines. The high humidity and salt fog characteristic of marine environments readily cause rapid corrosion of unprotected components. This advantage reduces component exposure time, lowers corrosion risks during the pretreatment stage, and simultaneously enhances construction efficiency, making it suitable for marine engineering rush projects and emergency repair scenarios. Additionally, the deposition process requires minimal target material consumption and features a straightforward procedure, significantly controlling material costs for large-scale coating applications. It also provides reference value for marine protective coating structures.

## Figures and Tables

**Table 1:** Research by Some Scholars on Coating Friction Corrosion.

Architecture	Deposition	Thickness	Environment	Wr (mm^3^·N^−1^·m^−1^)	References
N/Ti/TiN/GLC	MFMS	1.4 μm	Sea water	2.12 × 10^−6^	[24]
DLC	FCVA/HVP	5.75 μm	3.5 wt% NaCl	1.04 × 10^−6^	[8]
Cr/WC/a-C:H	UBMS	2.98 μm	Sea water	1.09 × 10^−7^	[27]
WC/a-C	HVOF/DCMS	1.7 μm	3.5 wt% NaCl	1.92 × 10^−7^	[6]
CrN/AlN	RMS	2.4 μm	Sea water	2.33 × 10^−7^	[31]

**Table 2:** Coating deposition parameters.

Sample	Layers	Ar (sccm)	C_2_H_2_ (sccm)	Time	Work Pressure	Bias Voltage
	Ti etching layer	200	0	300 s	0.5 Pa	−600 V
	Ti transition layer	200	0	1500 s	0.5 Pa	−100 V
S1	a-C	90	0	1320 s	0.2 Pa	−50 V
S2	a-C:H	90	10	1260 s	0.2 Pa	−50 V
S3	a-C (1)	90	0	660 s	0.2 Pa	−50 V
	a-C:H (1)	90	10	900 s	0.2 Pa	−50 V
S4	a-C (5)	90	0	132 s	0.2 Pa	−50 V
	a-C:H (5)	90	10	180 s	0.2 Pa	−50 V
S5	a-C (10)	90	0	71 s	0.2 Pa	−50 V
	a-C:H (10)	90	10	95 s	0.2 Pa	−50 V

**Table 3:** Arithmetic mean height ***Sa*** and three-dimensional root mean square roughness ***Sq*** of the sample.

Sample	HSLA100	S1	S2	S3	S4	S5
***S_a_*** (μm)	0.049 (±0.006)	0.066 (±0.008)	0.101 (±0.008)	0.177 (±0.010)	0.116 (±0.006)	0.091 (±0.007)
***S_q_*** (μm)	0.065 (±0.008)	0.091 (±0.01)	0.117 (±0.009)	0.185 (±0.012)	0.141 (±0.011)	0.117 (±0.009)
Grinding Mark Center ***S_a_*** (μm)	0.959 (±0.012)	0.499 (±0.011)	0.933 (±0.013)	0.267 (±0.010)	0.257 (±0.011)	0.106 (±0.009)
Grinding Mark Center ***S_q_*** (μm)	1.073 (±0.015)	0.503 (±0.013)	0.965 (±0.014)	0.268 (±0.012)	0.257 (±0.013)	0.113 (±0.009)

**Table 4:** The wear rate after dry friction test.

Sample	W_r_ (mm^3^·N^−1^·m^−1^)
HSLA100	2.09 (±0.20) × 10^−6^
S1	3.27 (±0.24) × 10^−7^
S2	3.12 (±0.26) × 10^−7^
S3	2.10 (±0.48) × 10^−7^
S4	1.88 (±0.16) × 10^−7^
S5	1.39 (±0.18) × 10^−7^

**Table 5:** The corrosion potential (E_corr_) and the corrosion current density per unit (i_corr_). (*n* = 3).

Sample	HSLA100	S1	S2	S3	S4	S5
E_corr_ (V)	−0.707	−0.439	−0.423	−0.375	−0.416	−0.437
i_corr_ (A/cm^2^)	1.43 (±0.06) × 10^−5^	2.76 (±0.09) × 10^−6^	2.59 (±0.05) × 10^−6^	2.46 (±0.07) × 10^−6^	2.41 (±0.04) × 10^−6^	2.15 (±0.09) × 10^−6^

**Table 6:** The wear rate exhibited by the samples after undergoing 1 h of tribocorrosion in 3.5 wt% NaCl solution.

Sample	Wr (mm^3^·N^−1^·m^−1^)
HSLA100	5.68 (±0.29) × 10^−5^
S1	7.59 (±0.72) × 10^−6^
S2	3.95 (±0.23) × 10^−6^
S3	1.15 (±0.07) × 10^−5^
S4	1.24 (±0.11) × 10^−5^
S5	4.53 (±0.02) × 10^−7^

## Data Availability

The data used to support the findings of this study are available from the corresponding author upon request.
